# Karyology of the Atlantic forest rodent *Juliomys* (Cricetidae): A new karyotype from southern Brazil

**DOI:** 10.1590/S1415-47572009005000031

**Published:** 2009-03-13

**Authors:** Roberta Paresque, Alexandre Uarth Christoff, Valéria Fagundes

**Affiliations:** 1 Departamento de Ciências Biológicas, Centro de Ciências Humanas e Naturais, Universidade Federal do Espírito Santo, Vitória, ESBrazil; 2 Museu de Ciências Naturais, Universidade Luterana do Brazil, Porto Alegre, RSBrazil

**Keywords:** * Juliomys*, karyotype evolution, *J. ossitenuis*, comparative G-banding, Ag-NORs

## Abstract

*Juliomys* is a small rodent from the family Cricetidae which inhabits the Atlantic forest and forests from Argentina to eastern Brazil. The three species recognized so far have different karyotypes. In this paper, we describe a new karyotype with 2n = 32, FN = 48 found in *Juliomys* specimens from a high-altitude area in the Atlantic forest of southern Brazil. The karyotype was analyzed after G- and C-banding and silver staining of the nucleolus organizer regions (Ag-NOR) and its G-banding patterns were compared with those of the newly described species *Juliomys ossitenuis* (2n = 20, FN = 36). The 2n = 32 karyomorph presented peculiar features and was very different from those of the other species of the genus: *J. pictipes* (2n = 36, FN = 34), *J. rimofrons* (2n = 20, FN = 34) and *J. ossitenuis* (2n = 20, FN = 36). Differences were mostly due to centric and tandem fusions, pericentric inversion and loss of heterochromatin. The karyotype represents a powerful tool to differentiate *Juliomys* species and our data suggest that the karyotype described herein belongs to a new species.

The family Cricetidae comprises five subfamilies (Arvicolinae, Cricetinae, Neotominae, Sigmodontinae and Tylomyinae) well-supported by molecular, anatomical and morphological data. Sigmodontinae is the most diverse, harboring nearly 380 species (55% of the Cricetidae) and 74 genera (57% of the Cricetidae). It is widespread in South America with representatives living throughout humid lowlands of the Amazon forest, Atlantic forest, subtropical savannah and high barren plains and in snow-capped peaks of the Andean Altiplano (Musser and Carleton, 2005).

The systematics and taxonomy of Sigmodontinae is still poorly understood and descriptions of new species (or even genera) have been recurrent (Weksler *et al.*, 2006) due to molecular, morphological or even karyological traits: *Akodonparanaensis* (Christoff *et al.*, 2000); *Juliomysrimofrons* (Oliveira and Bonvicino, 2002); *Oligoryzomysmoojeni* and *O. rupestris* (Weksler and Bonvicino, 2005); *Akodon philipmyersi* (Pardinãs *et al.*, 2005); and *J. ossitenuis* (Costa *et al.*, 2007). In some cases, new karyotypes were the first step in the identification of the new taxa, as *Akodon* sp. (Silva and Yonenaga-Yassuda, 1998) and *Calomys tocantinsi* (Fagundes *et al.* 2000; Bonvicino *et al.*, 2003).

*Juliomys* was recently described (González 2000) to accommodate the distinction between *J. pictipes* (Osgood 1933) and *Wilfredomys oenax* (Thomas 1928), small rodents characterized by having a tail longer than their head and body, long soft fur, short and broad feet, a pentalophodont molar, an ectolophid present in M_1_ and M_2_, a short rostrum in the skull, a wide braincase, and an anteriorly compressed zygomatic bone. The phylogenetic position of the genus in the family is still uncertain and Oliveira and Bonvicino (2002) proposed that *Juliomys* did not belong to any of Reigs eight tribes (Reig, 1984) according to morphological and DNA sequence data (cytochrome b). Phylogenetic analysis using the DNA sequence of the interphotoreceptor retinoid-binding protein (IRBP) exon placed *Juliomys* in a distinct clade as *incertae sedis* (Weksler, 2003; D'Elia *et al.*, 2006).

The three currently recognized species of *Juliomys* are distributed from Argentina to southeastern Brazil and up to Espírito Santo: *J. pictipes* (Osgood, 1933) has its type locality in Misiones (Argentina) and occurs in the Atlantic forest from São Paulo to Santa Catarina states in Brazil (Bonvicino and Otazu, 1999); *J. rimofrons*Oliveira and Bonvicino, 2002 is known exclusively from its type locality at Serra da Mantiqueira, municipality of Itamonte, state of Minas Gerais (Brazil); and *J. ossitenuis*Costa *et al.*, 2007 is found in broadleaf evergreen, semideciduous and montane forests above 800 meters, from Espírito Santo to southern São Paulo, in southeastern Brazil.

Conventional stained karyotypes are easily obtained from live animals and can be used for species distinction in *Juliomys* (Table 1): *J. pictipes* presents 2n = 36 and FN = 34 (Bonvicino and Otazu, 1999), *J. rimofrons* has 2n = 20 and FN = 34 (Oliveira and Bonvicino, 2002), and *J. ossitenuis* has 2n = 20, FN = 36 (Costa *et al.*, 2007). In a previous study, the conventionally stained karyotype of *J. ossitenuis* was described and compared with those of *J. rimofrons,* and *J. pictipes* (Costa *et al.*, 2007). Rearrangements such as centric and tandem fusions and pericentric inversions were involved in the karyotype differentiation within *Juliomys*. However, comparisons of G-banded karyotypes are required to the adequate identification of chromosome homologies and rearrangements, which, in turn, help in the understanding of the chromosomal evolution in the genus.

In this paper, we describe a new karyotype from *Juliomys* specimens collected in an area previously not sampled in southern Brazil and we describe for the first time the G- and C-banded karyotypes of *Juliomys*. In addition, we compared the new karyomorph of *Juliomys* with that of *J. ossitenuis* and we propose a hypothesis to explain the karyotype evolution in *Juliomys*.

Cytogenetic analysis was carried out on three males of *Juliomys* collected at Aparados da Serra National Park, municipality of São Francisco de Paula, state of Rio Grande do Sul, Brazil: MNCU464, MNCU868 and MNCU869, (29° 29' S and 51° 30' W, at 800 m altitude); and on two males of *Juliomys ossitenuis* from Caparaó National Park, municipality of Dores do Rio Preto, state of Espírito Santo, Brazil: MBML2784 (20° 46' S and 41° 81' W, at 2079 m altitude) and MBML2783 (20° 48' S and 41° 83' W, at 1788 m altitude). The specimens were deposited in the Museu de Ciências Naturais da Universidade Luterana do Brazil (MCNU), Canoas, Rio Grande do Sul, Brazil, and in the Museu de Biologia Prof. Mello Leitão (MBML), Santa Teresa, Espírito Santo, Brazil.

Chromosome preparations were obtained from bone marrow after *in vivo* injection of a 0.1% colchicine solution (1 mL/100 g of body weight). Cells were exposed to 0.075 M KCl for 25 min at 37 °C and subsequently fixed with Carnoy solution (methanol:acetic acid, 3:1). The cell suspensions were spread onto clean slides and air dried. GTG- and CBG-banding and Ag-NOR staining were performed according to Seabright (1971), Sumner (1972) and Howell and Black (1980), respectively. At least 20 metaphases per individual were analyzed to establish the diploid number (2n), the number of autosomal arms (FN) and the distribution pattern of the silver stained nucleolus organizer regions (Ag-NORs).

We found a new karyotype with 2n = 32 and FN = 48, with one pair of large submetacentrics (pair 1), eight pairs of biarmed autosomes with gradual variation in size (pairs 1-9) and six pairs of acrocentrics (pairs 10-15). The X chromosome was a large submetacentric (larger than chromosome 2), clearly distinct from the autosomes. The Y was a medium acrocentric, similar in size to chromosome 10 (Figure 1a). G-banding allowed the precise identification of all chromosome pairs (Figure 1c). C-banding revealed small blocks of constitutive heterochromatin at the pericentromeric regions of all autosomes, except for pair 1. Additional segments of heterochromatin were distally located in the long arms of pairs 6, 9 and 10. The X chromosome is entirely heterochromatic, except the distal long arm, as was the entire Y chromosome (Figure 1e). Four chromosome pairs had telomeric Ag-NORs (biarmed pairs 2 and 8; and acrocentric pairs 10 and 12). Three to seven Ag-NORs were observed per metaphase. Pairs 2, 8 and 12 showed a high frequency (80%-95%) of active NORs on the telomeres of the long arms of both homologues (2q, 8q, and 12q). Most metaphases only had Ag-NORs at one homologue of pair 10 (10q), which was less frequently labeled (40%-45%) than the other NORs (Figure 1g). We found inter and intraindividual variability of Ag-NORs.

*Juliomys ossitenuis* had 2n = 20 and FN = 36 with all biarmed chromosomes, except for the acrocentric Y chromosome (as previously described in Costa *et al.*, 2007 and illustrated in Figure 1b). The G-banding patterns are presented for the first time (Figure 1d) and the C-banding revealed constitutive heterochromatin at the centromeric region of most autosomes, in the long arm of the X and in the entire Y chromosome (Figure 1f). Multiple telomeric Ag-NORs were observed in five pairs: in the long arms of pairs 2 and 4 (2q and 4q), in the short arm of pair 3 (3p), and in the short arms of two medium biarmed pairs, not precisely identified, but probably pairs 5 and 6 (data not shown).

The comparative G-banding analysis (Figure 2) revealed homologies between whole chromosome arms of *J. ossitenuis* (JOS) and the new karyotype of *Juliomys* (JSP). Centric and tandem fusions, as well as pericentric inversions, frequently accounted for the differences between these karyotypes. Two chromosome pairs were completely conserved in the two species: the large submetacentric JSP1 showed complete correspondence with the metacentric JOS2, as was also the case for the medium submetacentrics JSP2 and JOS6. These homeologies were confirmed by the Ag-NOR location in the short arms of JSP2 and its homeologous JOS6. Four large biarmed JOS chromosomes corresponded to eight small JSP chromosomes. A pericentric inversion could explain the difference between the medium submetacentric JSP4 and the long arm of JOS1, while the acrocentric JSP14 corresponded to the short arm of JOS1. A pericentric inversion in the medium submetacentric JSP5 followed by a centric fusion with the acrocentric JSP15 would explain their correspondence with the long and short arm of the large submetacentric JOS3, respectively. A centric fusion between the acrocentrics JSP12 and JSP13 would account for their correspondence with the short and long arms of JOS5, respectively. Almost the entire JSP9 (except for the distal positive G-band in the long arm) corresponded to the short and proximal long arm of JOS4. The remaining portion of JOS4 corresponded to JSP10. The missing positive G- band of JSP9 corresponded to heterochromatin, as revealed by C-banding (Figure 1e). Thus, the loss of the telomere and heterochromatin of JSP 9 and of the centromere and telomere of JSP10, followed by the tandem fusion of JSP9 with JSP10 would have originated JOS4 and the centromeres of JSP9 and JOS4 should thus be homologous.

A comparative karyotypic analysis between *Juliomys* sp (2n = 32, FN = 48) and *J. pictipes* (2n = 36, FN = 34) based solely on conventional staining suggested that the complements differed by at least one centric fusion (CF) and eight pericentric inversions (PI). The karyotype of *J. ossitenuis* (with nine biarmed chromosome pairs) differs from that of *J. pictipes* (with 17 acrocentric pairs) by at least eight CFs plus one PI, while the complements of *J. ossitenuis* and *J. rimofrons* (eight biarmed and one acrocentric pair) differ by one PI. We thus suggest that eight CFs were involved in the karyotypic differentiation of *Juliomys pictipes* (2n = 36, FN = 34, Bonvicino and Otazu, 1999) and *Juliomys rimofrons* (2n = 20, FN = 34, Oliveira and Bonvicino, 2002).

The analyses of the karyotypes of *Juliomys* indicate that centric and tandem fusions plus pericentric inversions were the most frequent rearrangements involved in the karyotypic differentiation. Comparative G-banding analysis allowed us to conclude that the new karyotype of *Juliomys* sp and that of *J. ossitenuis* differed by centric fusions and by some complex rearrangements (tandem fusions, pericentric inversions, loss of heterochromatin). Similar chromosome rearrangements seem to have been involved in the karyotypic differentiation between *J. pictipes* and *J. rimofrons*.

The rearrangements between the karyotypes of different species are complex and involve transposition of whole chromosome segments, loss of chromosome segments and centromeres, as well as centric and tandem fusions. We thus believe that such rearrangements are enough to prevent individuals with different karyotypes from crossbreeding, acting as an effective postzygotic reproductive barrier. A similar mechanism has been documented in two cryptic and sympatric species of sigmodontine rodents: *Akodon cursor* (Winge, 1887) (2n = 14-16) and *A. montensis* Thomas, 1913 (2n = 24). Although there is almost complete correspondence between the chromosome arms of the two species which differ by pericentric inversion and tandem fusions (Fagundes *et al.*, 1997), hybrids are not viable (Yonenaga *et al.*, 1975). Moreover, specimens of *Juliomys* with different karyotypes are usually associated with different and disrupt geographic distributions, reinforcing their inability to crossbreed.

In a phylogenetic analysis based on the mitochondrial cytochrome b gene, Costa *et al.* (2007) concluded that each species of *Juliomys* could be grouped as a monophyletic clade with 10%-14% of divergence among them and each of these species presented a distinctive karyotype. Previous reports had already suggested that species-specific karyotypes were a characteristic of the genus *Juliomys* (Bonvicino and Otazu, 1999; Oliveira and Bonvicino, 2002).

In summary, the karyological uniqueness allied to the disruptive geographic distribution of the *Juliomys* specimens from southern Brazil herein studied indicate that it is a new species. Additional morphological and molecular studies are required for the formal species description and for conclusions about its phylogenetic position.

Costa *et al.* (2007) emphasized the urgent need for large scale collecting of small mammals even in supposedly “well-known” areas such as southeastern Brazil since biodiversity explorations in South America still frequently lead to the discovery of new taxa and the rediscovery of rare species (Percequillo *et al.*, 2004; Emmons and Patton, 2005; Weksler and Bonvicino, 2005). Species of *Juliomys* are never among the most common taxa at a given locality (Pardiñas *et al.*, 2005) and the largest series from a single site consists of just 11 (*J. pictipes)* or 28 *(Juliomys ossitenuis)* specimens. The Atlantic forest is one of the top biodiversity hotspots and it has been suffering from human induced habitat loss and fragmentation. Adequate efforts to catalog the biodiversity of this biome using ecological, morphological, molecular or karyological traits are vital for its conservation.

**Figure 1:**
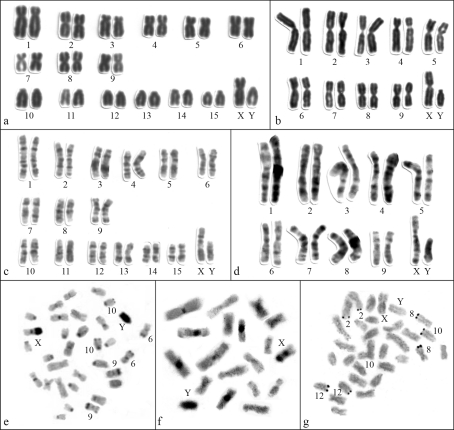
Karyotypes of (a, c, e) *Juliomys* sp (2n = 32; NF = 48) and of (b, d, f) *J*. *ossitenuis* (2n = 20; FN = 36) after: (a-b) conventional staining; (c-d) G-banding and (e-f) C-banding; (g) nucleolus organizer regions (Ag-NORs) in *Juliomys* sp (2n = 32, FN = 48). The numbers indicate the NOR-bearing chromosomes.

**Figure 2:**
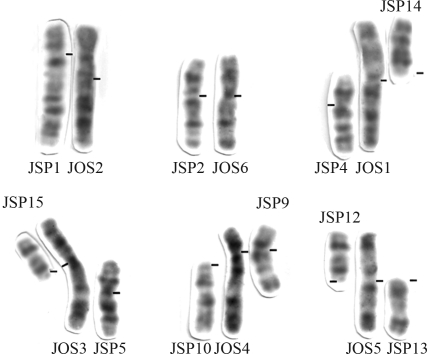
Comparison of the G-banded chromosomes of *Juliomys ossitenuis* (JOS) and of *Juliomys* sp (JSP). The bars indicate centromere positions.

## Figures and Tables

**Table 1:** Karyotypes of *Juliomys* species.

Species	2n	FN	Autosomes^1^		X^2^	Y^2^	Locality^3^	References
			M	A				
*J. pictipes*	36	34	-	17	A	A	SP, SC	González, 2000
*J. rimofrons*	20	34	8	1	SM	SM	RJ	Oliveira and Bonvicino, 2002
*J. ossitenuis*	20	36	9	-	M	A	SP, ES, RJ, MG	Costa *et al.*, 2007; present report
*Juliomys* sp.	32	48	9	6	SM	A	RS	Present report

2n = diploid number; FN = fundamental number. ^1^M = metacentric/submetacentric, A = acrocentric. ^2^M = metacentric, SM = submetacentric, A = acrocentric. ^3^States of Brazil: SP = São Paulo, SC = Santa Catarina, RJ = Rio de Janeiro, ES = Espírito Santo, RS = Rio Grande do Sul and MG = Minas Gerais.
